# Supplementary material for: Helicase Q promotes homology-driven DNA double-strand break repair and prevents tandem duplications
Source: Nat Commun. 2021 Dec 8;12:7126. doi: 10.1038/s41467-021-27408-z (PMC8654963; doi:10.1038/s41467-021-27408-z)
Supplement: Supplementary file 1 — Supplementary Information [file 41467_2021_27408_MOESM1_ESM.pdf]

Supplementary info

Helicase Q promotes homology-driven DNA double-strand break repair and prevents tandem duplication mutations

Kamp et al. 2021

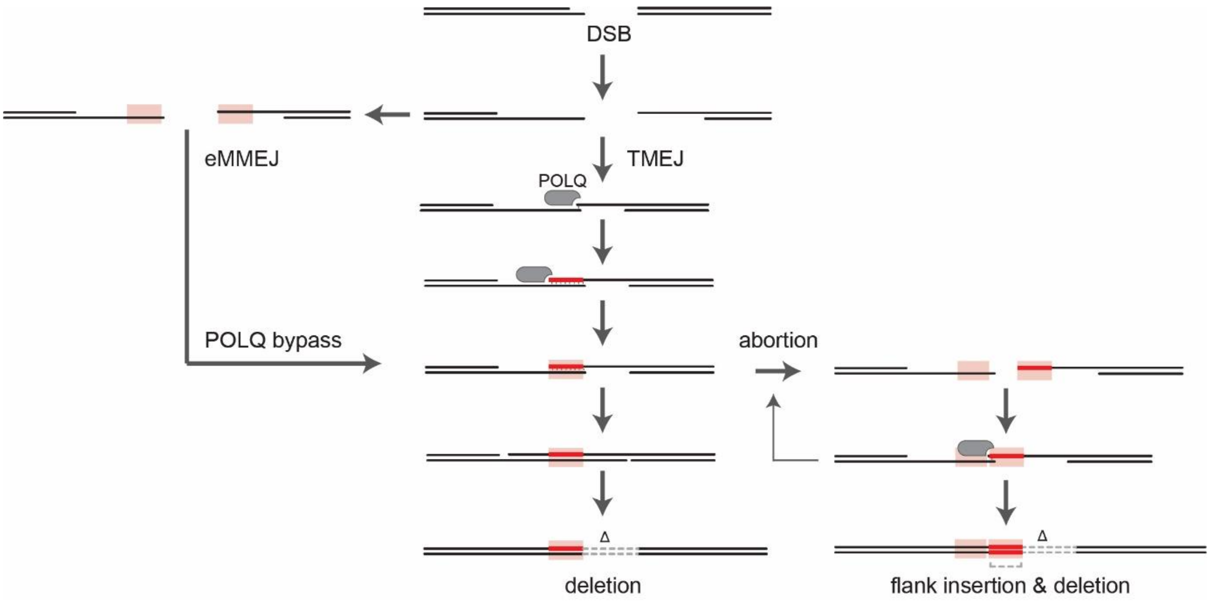

Supplementary Figure 1 Model illustrating i) how eMMEJ can bypass the requirement for POLQ in altEJ; ii) how abortive TMEJ results in templated insertions within deletion junctions.

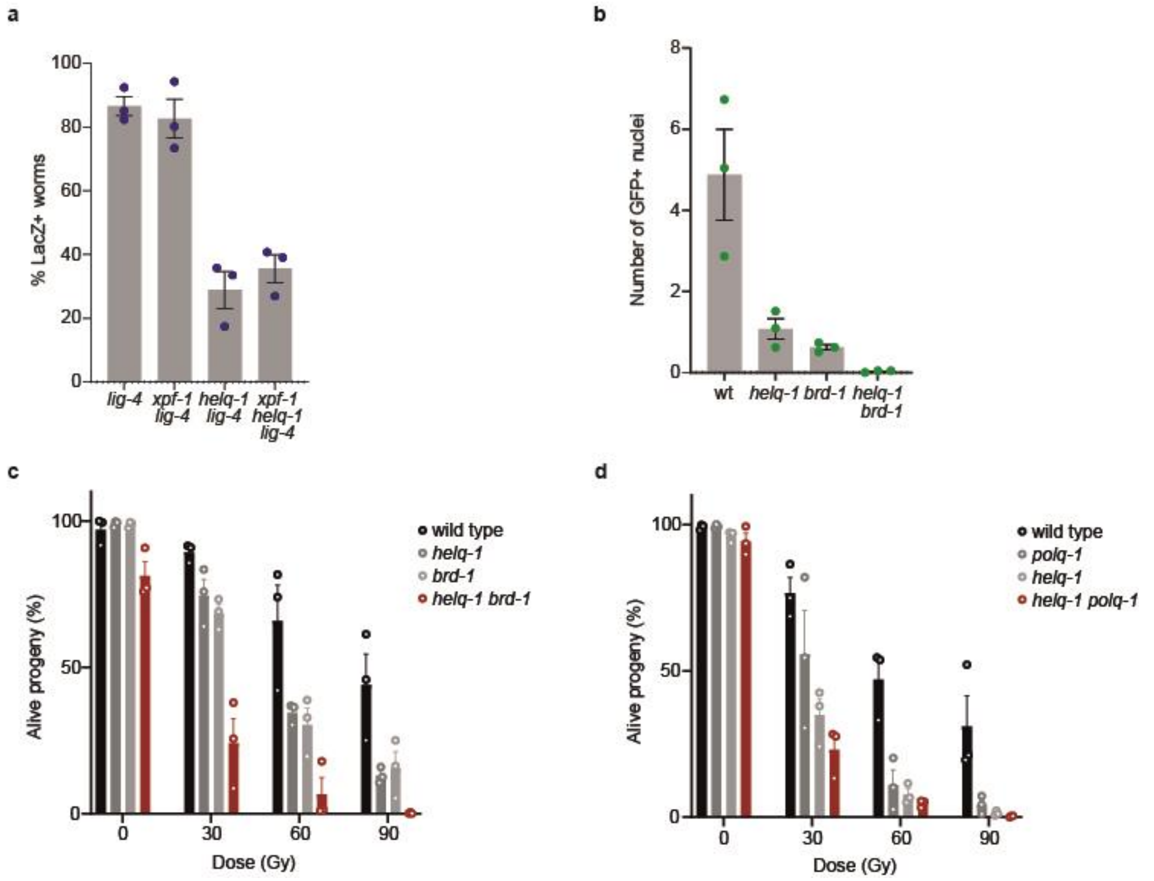

Supplementary figure 2 Effect of deficiency in POLQ-1 and HELQ-1 on sensitivity to ionizing radiation.

a. Histogram depicting the percentage of LacZ positive worms for the indicated genotype. Experiments were performed in triplicate. Each dot represents the average percentage of each replicate. Error bars represent SEM. b. Histograms depicting the average number of GFP expressing nuclei per worm for the indicated genotype. Experiments are performed in triplicate (n=45 per replicate). Each dot represents the average number of each replicate. Error bars represent SEM. c. Wild type (black), *helq-1* (grey), *brd-1* (light grey) and *helq-1 brd-1* (red) L4 larvae were exposed to 0, 30, 60 or 90 Gy IR (n = 3). Three days after irradiation, the numbers of alive offspring versus unviable eggs were quantified. The average survival in each experiment is represented in dots. Error bars represent SEM. d. Wild type (black), *polq-1* (grey), *helq-1* (light grey) and *helq-1 polq-1* (red) L4 larvae were exposed to 0, 30, 60 or 90 Gy IR (n = 3). Three days after irradiation, the numbers of alive offspring versus unviable eggs were quantified. The average survival in each experiment is represented in dots. Error bars represent SEM.

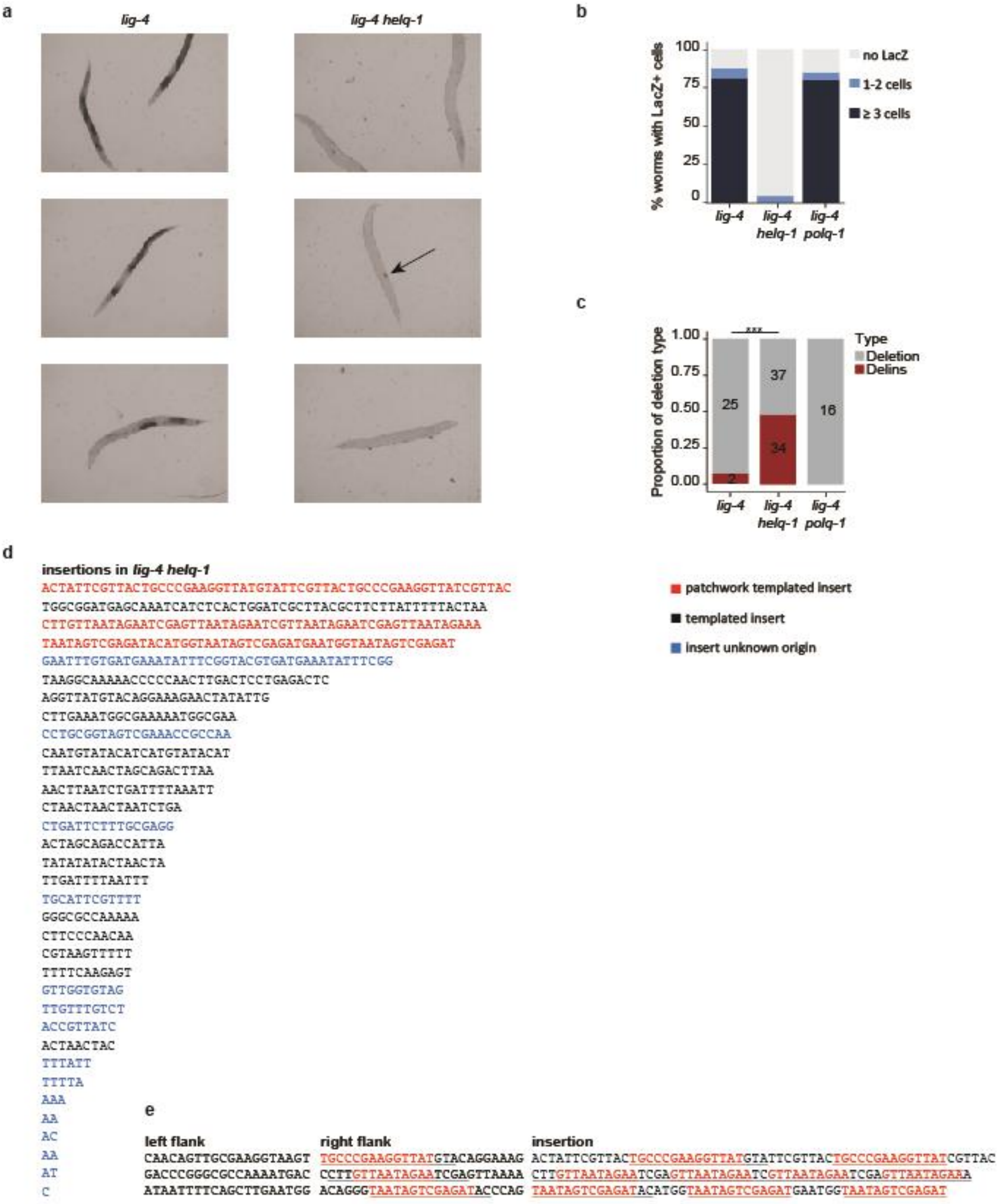

Supplementary figure 3 SSA deficiency and increased insertions in *C. elegans* lacking HELQ-1

a. Representative pictures of *lig-4* and *lig-4 helq-1* mutant animals carrying the reporter transgenes that were heat shocked to induce Isce-I expression, followed by staining for B-galactosidase expression. Arrow indicates staining scored as 1 LacZ+ cell. b. Histogram depicting the percentage of worms without LacZ+ cells, with one or two LacZ+ cells or more than two blue cells for the indicated genotype. Worms of three independent experiments were scored. c. Proportion of deletion types at the SSA reporter. Difference in ratio between deletions without and with insertion were tested

between *lig-4* and *lig-4 helq-1* using Chi square test (*** P<0.001). d. Sequences of DNA insertions identified within deletions at the SSA reporter in *lig-4 helq-1* animals (sorted to size). Patchwork insertions are represented in red, templated insertions in black, and insertions of which the origin could not be determined are represented in blue. e. Examples of deletions with patchwork insertions. For each case, the insertion and flanks of the corresponding deletion are depicted. Inserted nucleotide stretches that are identical to the flank of the deletion junction are underscored in both the insertion and flank. Nucleotide stretches present multiple times within one insertion are depicted in red.

a

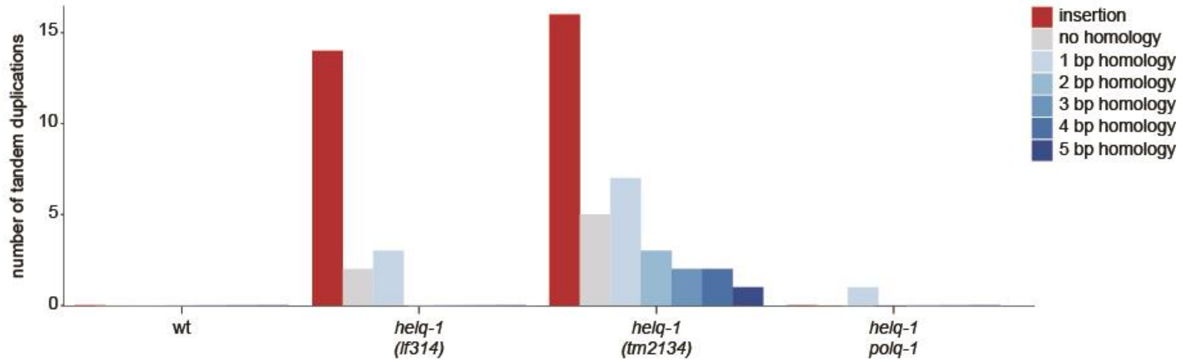

b

	insertion in TD
helq-1(lf314)	TTTCTTTGTCATCACTGTTTCTTTGTCACACTTGGCAGTTTCTTTGTCACACTTGGCAGTTTCTTTGTCACACT
helq-1(tm2134)	CACTGTTTTCACTGTTTTGAGCTTTCACTGTTTTCACTGTTTTTCACTGTTTCACTGCTTTCACTGTTTTG
helq-1(tm2134)	AAATTTGATAAATTTGATAATTATGTTTGATAATTATGTTTATTAAATTTGATAATTATGATAAATAAATA
helq-1(tm2134)	GGTATTCGCGTCTTAGTATTCGCATCTTGCTTATTGTATTCGCATCTTGCTT
helq-1(lf314)	CTGAAAACTTCTGAAAACCTTTCTGAAAAACTTTCTGAAAACCC
helq-1(tm2134)	GCATGTGTTCACATTGCAGACATGTGTTCACATTGCAGACTG
helq-1(tm2134)	CGTGTAACATGCAAGAAAATTAGAGAAAAAATTAAAAAT
helq-1(tm2134)	CAAGGGATACAAGGTACAAGGGATACAAGGTG
helq-1(tm2134)	CATCAAAAATCAGAAATCAAGAAATCAAAAA
helq-1(lf314)	AACTGACTCTCAAACTGGCTCTCTCTC
helq-1(tm2134)	ATTTTTTTTTTTTAAAATTTTTTTTTT
helq-1(tm2134)	AAAAACGATTCCATTTCAGGGATCGTT
helq-1(tm2134)	CAAACCAGTACATTTTCTTTTTTTTT
helq-1(tm2134)	AGTTTTTCCGTAAAAAAAAAAAAAAA
helq-1(lf314)	GAAATTTAAGGCTATTTAAAAAAA
helq-1(lf314)	GCATAACTTCAAGGAATGGCATA
helq-1(lf314)	CCTAGACTCTAGACCAGACC
helq-1(lf314)	GTCTGTAACAAGGCGAATG
helq-1(lf314)	ATGATACATATGCTGC
helq-1(lf314)	CTTATCTTTAGACTA
helq-1(tm2134)	AGAATGAAAAAAGAA
helq-1(lf314)	ATGTTTGTCAATT
helq-1(tm2134)	CTTTCGCAGAA
helq-1(lf314)	TTCATATTCA
helq-1(tm2134)	AAAGATGACT
helq-1(lf314)	TGTGGAG
helq-1(lf314)	AAAAAA
helq-1(tm2134)	TCTA
helq-1(tm2134)	TT
helq-1(lf314)	C

■ patchwork templated insert
■ patchwork insert unknown origin
■ templated insert
■ insert unknown origin

Supplementary figure 4 Mutational footprints of identified tandem duplications

a. Histogram depicting the different mutational footprints of tandem duplications that accumulated in genomes of indicated genotypes. Tandem duplications are colour-coded according to the following mutational classification: grey for simple deletions without apparent MH at the junction, blue for simple deletions with MH at the junction, wherein the saturation level of the blue colour increases with increasing amount of homology identified. Deletions containing insertions are depicted in red b. Insertions identified in tandem duplications that accumulated in genomes of *helq-1(lf314)* and *helq-1(tm2134)* animals. Insertions are sorted according to size. Patchwork templated insertions are represented in red, patchwork insertions of unknown origin in grey, simple templated insertions in black and insertions of which the origin could not be determined are represented in blue.

File	Genotype	Generations	Median Coverage
N2_2_60	Wildtype	60	34.38165
N2_3_60	Wildtype	60	57.39455
N2_4_60	Wildtype	60	18.4895
N2_50_60	Wildtype	60	26.0963
FX2134-D50	*helq-1(tm2134)*	50	36.62475
FX2134-G50	*helq-1(tm2134)*	50	43.48685
FX2134-I50	*helq-1(tm2134)*	50	39.7627
NL4716_50gen	*dog-1(pk2247)*	50	26.68345
NL4721_50gen	*dog-1(pk2247)*	50	9.99105
NL4722_50gen	*dog-1(pk2247)*	50	24.8927
XF1116_11_42	*dog-1(gk10) polq-1(tm2026)*	42	30.2128
XF1116_3_40	*dog-1(gk10) polq-1(tm2026)*	40	23.7144
XF1116_7_40	*dog-1(gk10) polq-1(tm2026)*	40	19.1549
XF1582-0	*helq-1(tm2134) polq-1(tm2026)*	0	37.10295
XF1582-B50	*helq-1(tm2134) polq-1(tm2026)*	50	43.79555
XF1582-E50	*helq-1(tm2134) polq-1(tm2026)*	50	43.77495
XF1582-G50	*helq-1(tm2134) polq-1(tm2026)*	50	36.13365
XF1700-0	*helq-1(lf314)*	0	29.9088
XF1700-50E	*helq-1(lf314)*	50	32.5171
XF1700-50H	*helq-1(lf314)*	50	34.87865
XF1700-50I	*helq-1(lf314)*	50	39.75315

Supplementary table 1 Mutation accumulation experiment sample characteristics Overview of the number of generations per sample and median sequencing depth per sample.

Locus	Primer function	Sequence
Qua213	External PCR forward	ctcagccaaggctacaaac
Qua213	External PCR reverse	ccggcaattacacatttgcc
Qua213	Internal PCR forward	gatacgtgtacatgaatagtc
Qua213	Internal PCR reverse	caaaactgtcgcctgacctc
Qua317	External PCR forward	cattgtgggaaaaatccgacg
Qua317	External PCR reverse	tttgccatcaaggttccagac
Qua317	Internal PCR forward	ctttagccaatttttgagcac
Qua317	Internal PCR reverse	ggatttcacagcgtcaagag
Qua915	External PCR forward	ccgttttataagcccaaaccg
Qua915	External PCR reverse	agtttccagtgaaaaatcgcg
Qua915	Internal PCR forward	tgcgcggttttgtgtgtgg
Qua915	Internal PCR reverse	agtttccagtgaaaaatcgcg
Qua1277	External PCR forward	cctgacaaacgcctactctc
Qua1277	External PCR reverse	ggggagaagccgcatccaa
Qua1277	Internal PCR forward	gaatcccttttaatttggcaatag
Qua1277	Internal PCR reverse	cacatggagacggagagaaac
SSA reporter	External PCR forward	cagacggaaaatgtatctggg
SSA reporter	External PCR reverse	agttcatccatgccatgtg
SSA reporter	Internal PCR forward	caaactataatcatctcactgg
SSA reporter	Internal PCR reverse	ggacagggccatcgccaattgg

Supplementary table 2 Primer sequences
