# Supplementary material for: Helicase Q promotes homology-driven DNA double-strand break repair and prevents tandem duplications
Source: Nat Commun. 2021 Dec 8;12:7126. doi: 10.1038/s41467-021-27408-z (PMC8654963; doi:10.1038/s41467-021-27408-z)
Supplement: Supplementary file 3 — Reporting Summary [file 41467_2021_27408_MOESM3_ESM.pdf]

nature portfolio

Corresponding author(s): Marcel Tijsterman

Last updated by author(s): Nov 12, 2021

Reporting Summary

Nature Portfolio wishes to improve the reproducibility of the work that we publish. This form provides structure for consistency and transparency in reporting. For further information on Nature Portfolio policies, see our Editorial Policies and the Editorial Policy Checklist.

Statistics

For all statistical analyses, confirm that the following items are present in the figure legend, table legend, main text, or Methods section.

n/a	Confirmed	
☐	☒	The exact sample size (*n*) for each experimental group/condition, given as a discrete number and unit of measurement
☒	☐	A statement on whether measurements were taken from distinct samples or whether the same sample was measured repeatedly
☐	☒	The statistical test(s) used AND whether they are one- or two-sided<br>*Only common tests should be described solely by name; describe more complex techniques in the Methods section.*
☒	☐	A description of all covariates tested
☒	☐	A description of any assumptions or corrections, such as tests of normality and adjustment for multiple comparisons
☐	☒	A full description of the statistical parameters including central tendency (e.g. means) or other basic estimates (e.g. regression coefficient) AND variation (e.g. standard deviation) or associated estimates of uncertainty (e.g. confidence intervals)
☐	☒	For null hypothesis testing, the test statistic (e.g. *F*, *t*, *r*) with confidence intervals, effect sizes, degrees of freedom and *P* value noted<br>*Give P values as exact values whenever suitable.*
☒	☐	For Bayesian analysis, information on the choice of priors and Markov chain Monte Carlo settings
☒	☐	For hierarchical and complex designs, identification of the appropriate level for tests and full reporting of outcomes
☒	☐	Estimates of effect sizes (e.g. Cohen's *d*, Pearson's *r*), indicating how they were calculated

Our web collection on statistics for biologists contains articles on many of the points above.

Software and code

Policy information about availability of computer code

Data collection	Image analysis, base calling and error calibration were performed using standard Illumina software: BCL output from the HiSeqX and Novaseq6000 platform was converted using bcl2fastq tool (Illumina, versions 2.17 to 2.20 have been used) using default parameters. Raw reads were mapped to the C. elegans reference genome (Wormbase release 235) by BWA (version 0.7.17) and further processed via SAMtools (1.10).
Data analysis	Pindel (0.2.5a8), Manta(1.6.0) and GRIDSS(2.8.0) were used for callling structural variations, mutations were visually inspected using IGV (2.8.3)

For manuscripts utilizing custom algorithms or software that are central to the research but not yet described in published literature, software must be made available to editors and reviewers. We strongly encourage code deposition in a community repository (e.g. GitHub). See the Nature Portfolio guidelines for submitting code & software for further information.

Data

Policy information about availability of data

All manuscripts must include a data availability statement. This statement should provide the following information, where applicable:

- Accession codes, unique identifiers, or web links for publicly available datasets
- A description of any restrictions on data availability
- For clinical datasets or third party data, please ensure that the statement adheres to our policy

Raw sequences have been made publicly available at NCBI SRA (accession code PRJNA718436, https://www.ncbi.nlm.nih.gov/bioproject/PRJNA718436). The N2 wild-type and dog-1 animals sequence data were published previously and can be found at NCBI SRA (accession codes PRJNA260487 https://www.ncbi.nlm.nih.gov/bioproject/ PRJNA260487 and PRJNA225882 https://www.ncbi.nlm.nih.gov/bioproject/225882SRP032440).

Field-specific reporting

Please select the one below that is the best fit for your research. If you are not sure, read the appropriate sections before making your selection.

- [x] Life sciences
- [ ] Behavioural & social sciences
- [ ] Ecological, evolutionary & environmental sciences

For a reference copy of the document with all sections, see nature.com/documents/nr-reporting-summary-flat.pdf

Life sciences study design

All studies must disclose on these points even when the disclosure is negative.

Sample size

For NGS experiments we know from previous publications that ~3*50 generations/genotype provides enough datapoints (>10 per genotype) to analyze (see: Van Schendel et al., Nat. Commun. 2015, DOI: 10.1038/ncomms8394 & Van Bostelen et al., PloS Genet 2020, DOI: 10.1371/journal.pgen.1008759), therefore we choose 3 lines as a minimum to sequence.

For the IR experiment, we know the required sample size from literature and previous work (e.g. Johnson et al, Plos Genet. 2012, DOI: 10.1371/journal.pgen.1003339). The sample sizes for the repair reporter data were also based on previous work (Johnson et al, Plos Genet. 2012, DOI: 10.1371/journal.pgen.1003339). Also sample sizes in the CRISPR/Cas9 experiment were chosen based on previous work (Van Schendel et al., Nat. Commun. 2015, DOI: 10.1038/ncomms8394).

For the PCR-based assays, we were able to identify statistically significant changes, indicating that our sample sizes were sufficient.

Data exclusions

No data was excluded

Replication

The PCRs at G4 loci were performed until sufficient data was collected. Three independent researchers collected the PCR data, collecting similar results: Bennie Lemmens started the PCR experiments on dog-1 and dog-1 polq-1, and his results were replicated by Juliette Kamp and later by Ron Romeijn.The data on dog-1 helq-1 were first collected by Juliette Kamp and later collected by Ron Romeijn. Therefore, al G4 loci data were independently replicated.
SSA and SDSA reporter experiments were performed independently three times, giving reproducible results.
The NGS experiment was replicated: We made a new helq-1 mutant to verify the tandem duplication phenotype, and replication was successful. This NGS experiment was performed independently.
In the CRISPR/Cas9 experiment, injection of worms with CRISPR/Cas9 injection mixes, phenotypic screening and follow-up using PCR was performed by three different researchers, who independently found reproducible results.
The IR sensitivity experiments were performed independently three times, giving reproducible results.

Randomization

Allocation was random: For each experiment, the first worm (in the right developmental stage for the experiment) that was seen through the microscope when the stock plate was observed was picked.

Blinding

Some experiments were blinded: the genotype and dose (if applicable) was not known when scoring in IR experiments and repair reporter experiments. In PCR assays, determining which bands were deletion bands was performed blind: bands were encircled on the gel image, in which the genotype of the PCRed sample was not visible. Phenotypic screening of injected progeny in the CRISPR/Cas9 was performed blind for the genotypes.
The mutation accumulation line experiment was not blinded, since the events were called by software and not scored by humans.

Reporting for specific materials, systems and methods

We require information from authors about some types of materials, experimental systems and methods used in many studies. Here, indicate whether each material, system or method listed is relevant to your study. If you are not sure if a list item applies to your research, read the appropriate section before selecting a response.

Materials & experimental systems

n/a	Involved in the study	
x		Antibodies
x		Eukaryotic cell lines
x		Palaeontology and archaeology
	x	Animals and other organisms
x		Human research participants
x		Clinical data
x		Dual use research of concern

Methods

n/a	Involved in the study	
x		ChIP-seq
x		Flow cytometry
x		MRI-based neuroimaging

Animals and other organisms

Policy information about studies involving animals; ARRIVE guidelines recommended for reporting animal research

Laboratory animals	Caenorhabditis elegans: hermaphrodites, L1 till adult stage, N2 background.
Wild animals	The study did not involve wild animals
Field-collected samples	The study did not involve field-collected samples
Ethics oversight	No ethical approval was required, since C.elegans is an invertebrate

Note that full information on the approval of the study protocol must also be provided in the manuscript.
