# Supplementary figures and images for: Helicase Q promotes homology-driven DNA double-strand break repair and prevents tandem duplications
Source: Nat Commun. 2021 Dec 8;12:7126. doi: 10.1038/s41467-021-27408-z (PMC8654963; doi:10.1038/s41467-021-27408-z)

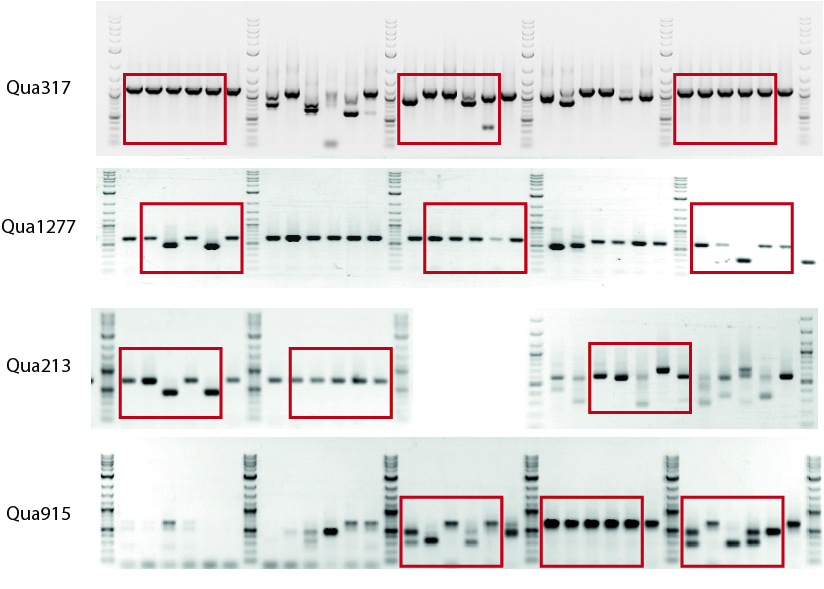

Supplement: Supplementary file 4 — Source Data [file 41467_2021_27408_MOESM4_ESM.zip › Kamp et al 2021 Source data/Source data gels Fig1.jpg]

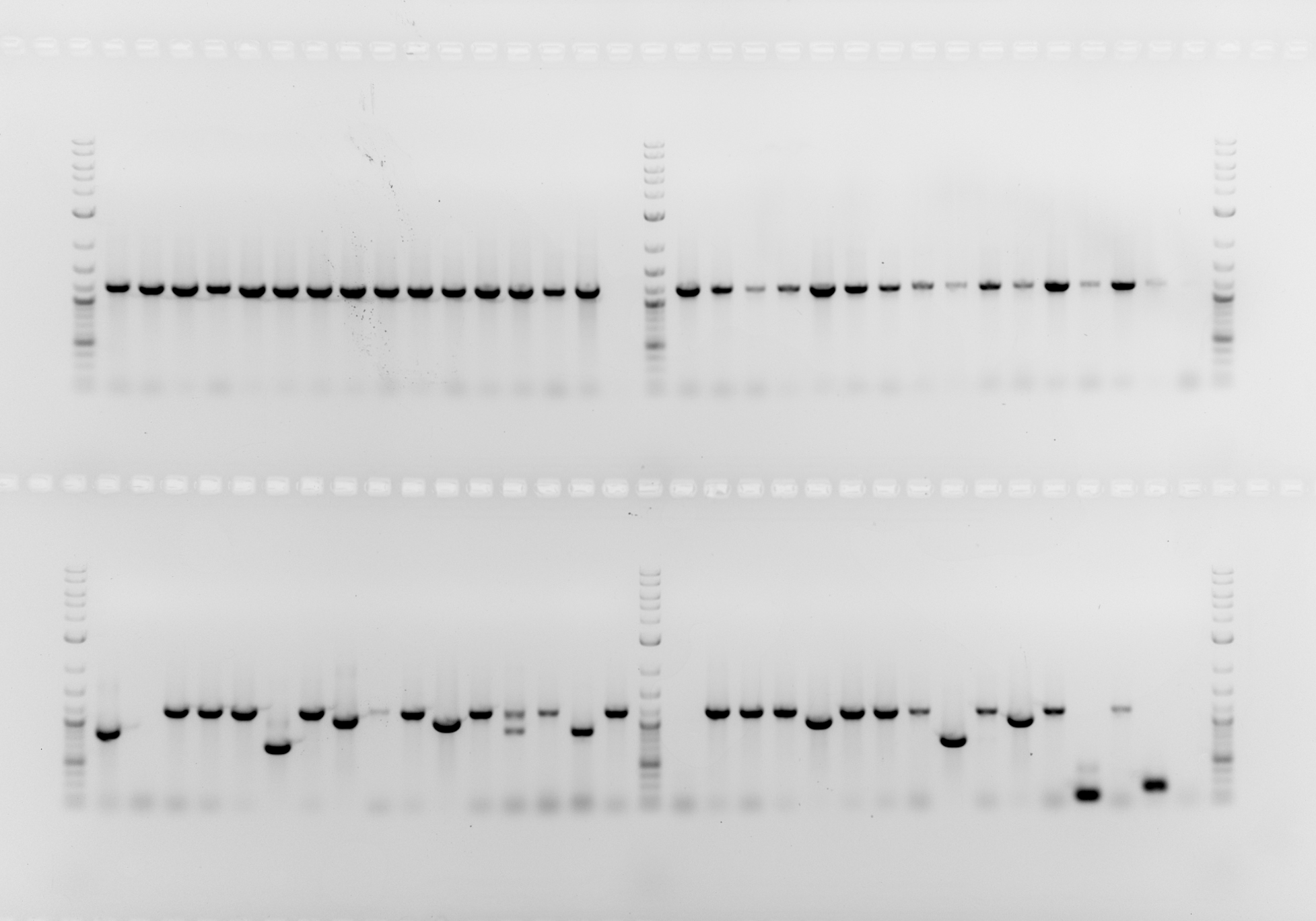

Supplement: Supplementary file 4 — Source Data [file 41467_2021_27408_MOESM4_ESM.zip › Kamp et al 2021 Source data/Source data Qua317 gel Fig 2.tif]

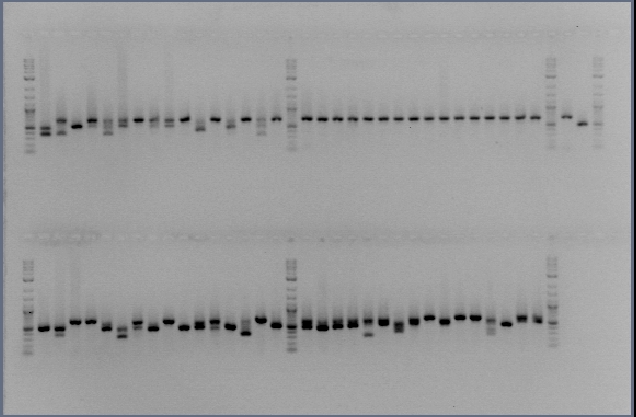

Supplement: Supplementary file 4 — Source Data [file 41467_2021_27408_MOESM4_ESM.zip › Kamp et al 2021 Source data/Source data Qua915 gel Fig2.jpg]
